# Investigation of the Demographic Characteristics and Mental Health in Self-Immolation Attempters

**DOI:** 10.5812/ijhrba.11159

**Published:** 2013-09-20

**Authors:** Seyedeh Narjes Zamani, Masoud Bagheri, Mohammad Abbas Nejad

**Affiliations:** 1 Department of Psychology, Shahid Bahonar University, Kerman, IR Iran; 2 Department of Foreign Languages, Shahid Bahonar University, Kerman, IR Iran

**Keywords:** Mental Health, Demographic Factors, Suicide

## Abstract

**Background:**

Self immolation is a heinous way to commit suicide which is mostly prevalent in individuals who attempt to escape a stressful situation and is considered as a strange and unusual method.

**Objectives:**

This study examined demographic characteristics and mental health in self immolation attempters in the city of Bandar Abbas.

**Materials and Methods:**

Two groups are involved in this enquiry. A group of 30 participants who have committed self-immolation and the other consisting of 15 non-committers. To conduct the study, MMPI questionnaire of Minnesota was employed accompanied with demographic information and descriptive statistics as well as independent “t-test”.

**Results:**

The research findings indicate that the self-immolation attempters were mostly among singles, females, low literates or illiterates, and housewives within the age group of 10-30 years old. According to the research there was a significant difference between two groups regarding personality characteristics and records of hypochondriasis, depression, psychopathic deviation, and hysteria. However no significant difference was observed between two groups regarding personality characteristics and paranoia, schizophrenia, psychasthrenia, and hypomania.

**Conclusions:**

Self-immolation should be considered as an important mental health problem by the authorities. Moreover, the government should plan to make families more aware in order to prevent self-immolation and also help to recognize the risk factors in this area.

## 1. Background

Suicide is death caused by one’s own intentional act, committed with full knowledge and expectation of death (1). Suicide is a psychosocial problem, at present on the rise due to the increasing complications of interactions and communications in most societies (2). One of the most tragic methods of suicide is self-immolation which maintains higher prevalence in the East rather than the West (3).

Mostly prevalent in individuals who attempt to escape a stressful situation (4), self-immolation is considered as a strange and unusual method in Euro-American culture (5). People commit self-immolation mostly due to different social and economic reasons and as political protest (6). The majority of attempters of self-immolation have records of previous suicide attempts and psychological disorders such as personality disorders, schizophrenia, economic and social problems (7). In a study in 2004, Roui concluded that among other psychological disorders mood disorders was associated with the highest suicide risk in both males and females (8). Hares and Baraclarec maintain that the highest suicide rate was observed in subjects suffering from dysthymia (12%) and major depressive disorder (20%) (9). In a study by Donald and Cameron during 1990 to 1995 on 1072 subjects who referred due to burn, 40 of which had attempted self-immolation, it was concluded that Schizophrenia, depression, and personality disorder were diagnosed in 71% of them (10). Goodwin and Marzic (11) reported a significant relationship between psychological disorders and suicide attempts in their study in the US. In a study on the characteristics related to suicide attempts, Lopez and Cast Roman et al. concluded that the most prevalent causes of suicide attempts are mood disorders, anxiety disorders and alcohol use disorders (12). Fridman and Asmeit declared that 17% of suicide committers suffered from panic disorder, 9% from other anxiety disorders, 33% from Schizophrenia, and 40% from depression (13). A 10-year study in Verona on 31 committers of self-immolation by Castellani and Beghini revealed that most subjects had records of psychological problems (7). Among theories concerning suicide, theories of Durkheim, Freud, and Menninger, can be noted among others. Nowadays, however, some people may not accept that a specific psychological dynamics or personality structure is associated with suicide. Regarding suicides there are a number of risk factors, including psychiatric disorders, social, cognitive, biological factors, and physical illnesses. Schizophrenia, mood disorders, especially depression, personality disorders, and panic disorders are considered as predictive of suicide (14).

Due to the fact that self-immolation causes facial contortion and sometimes physical impairment, rate of recurrent suicidal attempts rises, especially in psychiatric patients (15). To summarize, previous studies have shown that familial and environmental factors may cause self-immolation. Psychological changes, on the other hand, may also push individuals towards self-immolation. Furthermore, subjects who have committed self-immolation and are now suffering from the side effects, face an increased risk of recurrent suicide attempts. The necessity of conducting scientific studies to identify the different aspects of this biological-psychological and social phenomenon seems evident accordingly. The Burns Ward of Shahid Mohammadi Hospital of Bandar Abbas is considered the most important burn center across Hormozgan Province, Iran, to which all the cases of immolation, including self-immolation, throughout the province are referred. The present study was conducted on all the referring patients of self-immolation, within a one-year period from June 2011 to June 20, 2012.

## 2. Objectives

This study examined demographic characteristics and mental health in self immolation attempters in the city of Bandar Abbas.

## 3. Materials and Methods

The present research was conducted as a descriptive-analytical study. The statistical population included all the cases of self-immolation referred to Shahid Mohammadi Hospital of Bandar Abbas from June 2011 to June 20, 2012. The final sample included 30 committers of self-immolation, selected through available sampling and with regard to the objectives of the study, who were compared with 30 non-committer among the accompaniers and patients of the Burns Ward clients included via random sampling who were matched with group one in terms of age, gender, and place of residence. The demographic data including age, gender, record of opioids use, physical problems, level of education, and marital status were initially collected. Then, the mental condition of the patients as well as that of the peer group was examined through interviews, adopting MMPI questionnaire (short form). Independent t-test was employed for inferential data analysis. It was ensured that the participants consented consciously to the sampling and participation in the study. They were ultimately thanked for their cooperation and were ensured of the data confidentiality. Moreover, the pre-assumption of normal distribution of validity and clinical variables was assessed using the Kolmogorov–Smirnov test, which was established regarding all the variables in the two groups (P > 0.05). The pre-assumption of equality of variances in both groups was assessed using Levene’s test in three validity scales and eight clinical scales in the level (P > 0.05). All the required information were confidentially collected and recorded and the interviews were conducted on the condition of the patients’ consent. The MMPI short form contains 71 questions, the reliability of which was reported by Okhovat and Daneshmand (16) through calculation of Cronbach's alpha coefficient of 78% for the entire test. Numerous studies have been conducted concerning the validity of this test. Sheppard et al. examined the structural validity of this test and deemed it as desirable. Out of the total 11 standard scales of the test under discussion, three scales pertain to validity scales, namely correction, infrequency, lie, and the other eight scales pertain to clinical or personality criteria, namely, hysteria, depression, hypochondriasis, hypomania, schizophrenia, psychasthenia, paranoia, and psychopathic deviation.

## 4. Results

According to Table 1, 60% (18) of self-immolation committers were female, and 40% (12) were male. Of the subjects, 60% (18) were single, 33.30% (10) were married, and 6.70% (2) were divorced. As for the level of education, 26.70% (8) were as shown further in Table 2, 40% (12) were housewives, 7.6% (2) were employed, 13.3% (4) were students, 13.3% (4) were unilliterate, 36.70% (12) had elementary education, and 40% (10) had high school education; none of the subjects had academic education. Furthermore, 40% (12) were in the age range of 10-20 years, 40% (12) in the age range of 21-30 years, 6.7% (2) in the age range of 31-40 years, 6.7% (2) in the age range of 41-50 years, and 6.7% (2) were in the age range of 51-60 years old.

**Table 1.** Demographic Characteristics of Self-Immolation Attempters

	Self-Immolation, No. (%)
**Gender**	
Male	12 (40)
Female	18 (60)
**Age Range, year**	
10-20	12 (40)
21-30	12 (40)
31-40	2 (6.7)
41-50	2 (16.7)
51-60	2 (6.7)
**Marital Status**	
Single	18 (60)
Married	10 (33.3)
Divorced	2 (6.7)
**Level of education**	
Illiterate	8 (26.7)
Elementary	12 (40)
High School	10 (33.3)
University	0

**Table 2.** The Demographic Characteristics of Self-Immolation Attempters

	Self-Immolation, NO. (%)
**Occupation**	
Housewife	12 (40)
Employed	2 (6.7)
Student	4 (13.3)
Unemployed	4 (13.3)
Self-Employed	8 (26.7)
**Cause**	
Family problems	13 (43.3)
Conjugal fight	3 (10)
Financial problems	4 (13.3)
Physical illness	5 (16.7)
Romantic relationships	5 (16.7)

**Table 3.** Average, Standard Deviation, t-Score, Degree Of Freedom and the Approved Alpha Concerning the Mental Condition of Non-Committers and Self-Immolation Committers Admitted to the Burns Ward Of Shahid Mohammadi Hospital of Bandar Abbas In 2011-2012

	No.	Average	Standard Deviation	t	df	P Value
**Hypochondriasis**						P < 0.05
Committer	30	6.73	2.504	3.67	43	
Non-committer	15	4.07	1.792	4.00	43	
**Depression**						P < 0.0001
Committer	30	10.53	3.84	5.819	43	
Non-committer	15	4.60	1.12	7.808	43	
**Hysteria**						P < 0.0001
Committer	30	11.53	3.003	7.768	43	
Non-committer	15	5.20	1.320	9.811	43	
**Psychopathic Deviation**						P < 0.05
Committer	30	10.47	2.93	7.669	43	
Non-committer	15	4.40	1.18	9.840	43	
**Paranoia**						P > 0.05
Committer	30	7.50	1.907	5.784	43	
Non-committer	15	4.00	1.964	5.690	43	
**Psychasthenia**						P > 0.05
Committer	30	9.93	3.676	4.719	43	
Non-committer	15	5.00	2.360	5.442	43	
**Schizophrenia**						P > 0.05
Committer	30	11.30	2.654	8.034	43	
Non-committer	15	5.40	1.404	9.749	43	
**Hypomania**						P > 0.05
Committer	30	5.80	1.919	3.268	43	
Non-committer	15	3.87	1.767	3.360	43	

Employed and 26.7% (8) were self-employed. Findings listed in Table 2, portray that family problems at a rate of 43.3% (13) proved to be the most prevalent cause for self-immolation, followed respectively by 16.7% (5) due to physical illness, 16.7% (5) due to romantic relationships, 13.3% (4) due to financial problems, and 10% (3) due to conjugal fights.

The results of independent t-test for the two self-immolator and non-committer groups showed that the hypothesis was rejected, i.e. there is no significant difference between the two groups concerning personality characteristics. As indicated by the distribution of averages, there is a significant difference (P < 0.05) between the degree of Hypochondriasis in the committers of self-immolation (M: 6.73; SD: 2; t: 3.67) and that of non-committers (M: 4.07; SD: 1.79; t: 4.00). There was also a significant difference (P < 0.0001) between the degree of depression in the non-committers (M: 4.60; SD: 1.12; t: 7.80) and that of self-immolation committers (M: 10.53; SD: 3.84; t: 5.81). The distribution of averages furthermore revealed that hysteria grades of self-immolation committers (M: 11.53; SD: 3.00; t: 7.76) were significantly higher than those of non-committers (M: 5.20; SD: 1.32; t: 9.81) (P < 0.0001). As for psychopathic deviation, there was a significant difference (P < 0.05) between the committers (M: 10.47; SD: 2.93; t: 7.66) and non-committers (M: 4.40; SD: 1.18; t: 9.84), while, concerning paranoid results, there was no significant differences (P > 0.05) between the self-immolation committers (M: 7.50; SD: 1.90; t: 5.78) and non-committers (M: 4.00; SD: 3.67; t: 4.71). Also, regarding psychasthenia, no significant difference (P > 0.05) was observed between self-immolation committers (M: 9.93; SD: 1.90; t: 5.78) and non-committers (M: 5.00; SD: 2.36; t: 5.44). As was the case for schizophrenia and hypomania results with no significant difference for self-immolation committers (M: 11.30; SD: 2.65; t: 8.03) and (M: 5.80; SD: 1.91; t: 3.26) and non-committers (M: 5.40; SD: 1.40; t: 9.74) and (M: 3.87; SD: 1.76; t: 3.36) respectively at (P > 0.05).

## 5. Conclusions

According to the results, the majority of the examined individuals, i.e. 53.3%, were in the age group of 10-30 years with elementary and below education, 60% of whom were single which met the presented statistics by similar studies. In a 4-year long study by Maghsoudi et al. 99% of the committers of self-immolation were females of 25.5 average age, the majority of whom were housewives, illiterate, and economically disadvantaged (17). Rezaie et al. in another 4-month long study in Kermanshah reported that 62.5% of suicide committers were female, 57.8% were married, and 43.8% were in the age group 15-24 years (18). A 10-year study by Sheth et al. reported that the most incident suicide attempts were in females (19). Similarly, Zatghami and Khalilian in another study in Mazandaran declared that most committers were in the age group of 14-30 years, single, and low literate. As was the case with the study by Amirmoradi et al. who reported that the most cases of self-immolation committers in the age group of 21-25 years and low literate (15).

In their 2-year epidemiological study on self-immolation, Laloe and Ganesan reported that 79% of the victims were female in the age group of 15-34 years and concluded that the most prevalent cause of self-immolation to be conjugal problems (20). However, studies conducted in different parts across the globe have reached different findings. As stated by Poeschla and Combs subsequent to their study from 1973 to 2010, epidemiological criteria as well as psychological risk factors and methods of committing self-immolation are different in high versus low income countries. Self-immolation in low income countries is most prevalent in females and associated with psychiatric records, whereas, in high income countries the majority of committers of self- immolation are male (21). Furthermore, according to the conducted studies the most prevalent causes of self-immolation are reported to be family problems 40%, physical illnesses 16.6%, romantic relationships 16.6%, financial issues 13.3%, and conjugal fights 10%. As concluded by Amirmoradi et al. (22), 93.4% of women experienced their husbands’ physical and verbal abuse, 60% of which deemed their husbands as the main instigators of self-immolation. Subsequent to a 10-year investigation on 46 cases of self-immolation (36 males and 11 females) from 1990 to 2000, Rothschild and Raatschen introduced separation and financial problems as the main causes of self-immolation, and found that 65% of the cases had records of psychological disorders (23). Makhlouf and Alvarez, on the other hand, reported the main cause of self-immolation as emotional and financial problems, based on 18 years of investigating suicide attempts. Among the cases studied by Rezaei and Farzaneh, 29.7% had records of psychological illness, 15.6% had records of chronic physical illness, and four cases suffered from both. Out of the 31 participants in a 10-year retrospective study by Castellani and Beghini, six cases had records of opioids dependency and three suffered from Aids (7). Palmu and Isometsa et al. in an 8-year retrospective study in Finland in 2004 (24), as well as Michael in a worldwide study on self-immolation declared the most prevalent causes of self-immolation as to be consequences of unemployment and family internal conflicts (25). The findings of the present study showed that there was a significant difference between the two groups in terms of the degree of characteristics of depression, hypochondriasis, hysteria, and psychopathic deviation which conforms to the results of similar studies. According to Lopez and Castromani et al. the most prevalent causes of suicide attempts are mood disorders, anxiety disorders, and alcohol and opioids abuse disorders. In their study, Donald and Cameron maintained that 71% of the studied cases suffered from schizophrenia, depression, and personality disorder (26). García-Sánchez, Palao & Legarre reported that 63% of the studied cases suffered from one psychiatric disorder (3). In a 5-year study in north-western Thames, Davidson concluded that more than 3.4 of attempters of suicide had records of psychological disorder.
